# Reply to Pettersson et al.: Why X-ray spectral features are compatible to continuous distribution models in ambient water

**DOI:** 10.1073/pnas.1909551116

**Published:** 2019-08-20

**Authors:** Johannes Niskanen, Mattis Fondell, Christoph J. Sahle, Sebastian Eckert, Raphael M. Jay, Keith Gilmore, Annette Pietzsch, Marcus Dantz, Xingye Lu, Daniel E. McNally, Thorsten Schmitt, Vinicius Vaz da Cruz, Victor Kimberg, Faris Gel’mukhanov, Alexander Föhlisch

**Affiliations:** ^a^Helmholtz Zentrum Berlin für Materialien und Energie GmbH, Institute for Methods and Instrumentation for Synchrotron Radiation Research, D-12489 Berlin, Germany;; ^b^Department of Physics and Astronomy, University of Turku, FI-20014 Turun Yliopisto, Finland;; ^c^European Synchrotron Radiation Facility, F-38043 Grenoble, France;; ^d^Institute of Physics and Astronomy, University of Potsdam, 14476 Potsdam, Germany;; ^e^Swiss Light Source, Photon Science Division, Paul Scherrer Institut, 5232 Villigen PSI, Switzerland;; ^f^Theoretical Chemistry and Biology, Royal Institute of Technology, SE-10691 Stockholm, Sweden;; ^g^Institute of Nanotechnology, Spectroscopy and Quantum Chemistry, Siberian Federal University, 660041 Krasnoyarsk, Russia

“Ambient water properties have been shown to require heterogeneity” (1) is the imperative followed by Pettersson et al. (2) to relate X-ray spectroscopic findings to a heterogeneous or 2-phase model of ambient water. In ref. 3 we question this hypothesis based on quantitative X-ray spectroscopic evidence. We come to conclude that X-ray spectroscopies support no observations related to heterogeneous, distinct structural motives in ambient water.

The critique of sum rules by Pettersson et al. (2) is unjustified: Through normalization to the asymptotic behavior we avoid sum rule normalization of X-ray absorption spectroscopy (XAS). In liquid water, extended X-ray absorption fine structure oscillations are less than a percent for the used normalization range (4) (digitized), and less than twice that for ice (5).

Pettersson et al. (2) point toward well-known discrepancies between XAS calculations and experiments. For that reason, our analysis is not based on exact reproduction of intensity ratios. However, the used linear dependence of preedge peak intensity is not empirical as claimed by Pettersson et al., but based on first-principles simulations, outlined in ref. 3.

The occurrence of different vibrations in connection to different water molecules in the liquid was carefully quantified in our recent article (6), whereas a quantitative analysis is completely missing from ref. 7. Strong asymmetries in the hydrogen bond donation result in mixing of the states of b_1_, b_2_, and a_1_ symmetries (8), which has been found to explain resonant inelastic X-ray scattering asymmetry experiments with contemporary ab initio molecular dynamics simulations (9).

Pettersson et al. (2) criticize the comparative discussion to phases of ice (reference 46 in ref. 3). We point out similarities to ambient liquid water and set attention to reference 46 in ref. 3 where high-temperature ice spectra were reported to vary notably from sample to sample. We also point out that our simulated instantaneous X-ray emission spectra (XES) for core-hole dynamics agree with others (e.g., ref. 8). The fact that peak B′ maintains position in time-integrated and site-averaged spectra reflects the remnant unpropagated fraction of the evolving ensemble following core ionization. Correct relative energy alignment of the XES calculations at different times and sites is accomplished by evaluating the chemical shifts as in ref. 10.

Local structural motifs and assignments from high- and low-density liquids (HDL and LDL) have been claimed to motivate X-ray spectral features (e.g., ref. 7). We show in ref. 3 that X-ray spectral features are compatible with continuous distribution models. Our findings (3) might resonate with English and Tse on size effects (11): HDL and LDL, as concepts based on density, have thermodynamical meaning only for volumes larger than approximately 1 nm^3^ (11). In these applicable size scales “the density of bulk water at ambient conditions is homogeneous” (11).
